# Fourier series of finite products of Bernoulli and Genocchi functions

**DOI:** 10.1186/s13660-017-1431-y

**Published:** 2017-06-30

**Authors:** Taekyun Kim, Dae San Kim, Gwan-Woo Jang, Jongkyum Kwon

**Affiliations:** 1 Department of Mathematics, Kwangwoon University, Seoul, 139-701 Republic of Korea; 2 Department of Mathematics, College of Science, Tianjin Polytechnic University, Tianjin, 300160 China; 3 Department of Mathematics, Sogang University, Seoul, 121-742 Republic of Korea; 4 Department of Mathematics Education and RINS, Gyeongsang National University, Jinju, Gyeongsangnamdo 52828 Republic of Korea

**Keywords:** 11B68, 11B83, 42A16, Fourier series, finite product, Bernoulli function, Genocchi function

## Abstract

In this paper, we consider three types of functions given by products of Bernoulli and Genocchi functions and derive some new identities arising from Fourier series expansions associated with Bernoulli and Genocchi functions. Furthermore, we will express each of them in terms of Bernoulli functions.

## Introduction

Utilizing the generating function, the Bernoulli polynomials $B_{m}(x)$ can be written as

1.1 $$ \frac{t}{e^{t} - 1} e^{xt} = \sum _{m=0} ^{\infty} B_{m} (x) \frac {t^{m}}{m!} \quad{\text{(see [1--5])}}. $$

For $x=0$, $B_{m}=B_{m}(0)$ are called Bernoulli numbers.

As a second definition, we have the Genocchi polynomials $G_{m}(x)$ by the generating function as follows:

1.2 $$ \frac{2t}{e^{t}+1} e^{xt} = \sum _{m=0} ^{\infty} G_{m} (x) \frac {t^{m}}{m!} \quad{\text{(see [6--10])}}. $$

For $x=0$, $G_{m}=G_{m}(0)$ are called Genocchi numbers.

As to the Bernoulli and Genocchi polynomials and numbers, we will need only the following:

1.3 $$ \begin{aligned} & \frac{d}{dx} B_{m}(x) = m B_{m-1}(x) ,\qquad \frac{d}{dx} G_{m}(x) = m G_{m-1}(x) \quad(m \geq1), \\ & B_{m}(1) = B_{m} + \delta_{1,m},\qquad G_{m}(1) = -G_{m} + 2\delta_{1,m} \quad(m \geq0), \\ & G_{0}(x) = 0 ,\qquad \deg G_{m} (x) = m-1 \quad(m \geq1). \end{aligned} $$

For any real number *x*, we let

1.4 $$ \langle x\rangle = x - [x] \in[0,1) $$

denote the fractional part of *x*.

We recall the following facts about the Fourier series expansion of Bernoulli functions $B_{m}(\langle x\rangle)$:

(a) for $m \geq2 $,

1.5 $$ B_{m}\bigl(\langle x\rangle\bigr) = - m! \sum _{n= - \infty, n \neq0}^{\infty} \frac{e^{2\pi i nx}}{(2\pi i n)^{m}}, $$

(b) for $m = 1 $,

1.6 $$ - \sum_{n= - \infty, n \neq0}^{\infty} \frac{e^{2\pi i nx}}{2\pi i n} = \textstyle\begin{cases} B_{1}(\langle x\rangle),& \textrm{for } x\notin\mathbb{Z},\\ 0,&\textrm{for } x\in\mathbb{Z}. \end{cases} $$

Throughout this paper, we will assume that *r* and *s* are nonnegative integers with $r+s \geq1$. Here we will consider three types of sums of finite products of Bernoulli and Genocchi functions $\alpha_{m}(\langle x\rangle)$, $\beta_{m}(\langle x\rangle)$, and $\gamma_{m}(\langle x\rangle)$ and derive the Fourier series expansions of them. In addition, we will express each of them in terms of Bernoulli functions. We have

$$\begin{aligned}& (1)\quad \alpha_{m}\bigl(\langle x\rangle\bigr)=\sum _{c_{1} + \cdots+ c_{r} + j_{1} + \cdots+ j_{s} = m} B_{c_{1}}\bigl(\langle x\rangle\bigr)\cdots B_{c_{r}}\bigl(\langle x\rangle\bigr) \times G_{j_{1}}\bigl(\langle x\rangle\bigr)\cdots G_{j_{s}}\bigl(\langle x\rangle\bigr)\quad (m > s); \\& \begin{aligned}[b] (2)\quad\beta_{m}\bigl(\langle x\rangle \bigr)&=\sum_{c_{1} + \cdots+ c_{r} + j_{1} + \cdots+ j_{s} = m} \frac{1}{c_{1}! \cdots c_{r}! j_{1}! \cdots j_{s}!} \\ &\quad{}\times B_{c_{1}}\bigl(\langle x\rangle\bigr)\cdots B_{c_{r}}\bigl(\langle x\rangle\bigr)G_{j_{1}}\bigl(\langle x \rangle\bigr)\cdots G_{j_{s}}\bigl(\langle x\rangle\bigr) \quad(m > s); \end{aligned} \\& \begin{aligned}[b] (3)\quad\gamma_{m}\bigl(\langle x\rangle \bigr)&= \sum_{c_{1} + \cdots+ c_{r} + j_{1} + \cdots+ j_{s} = m} \frac{1}{c_{1} \cdots c_{r} j_{1} \cdots j_{s}}B_{c_{1}} \bigl(\langle x\rangle\bigr)\cdots B_{c_{r}}\bigl(\langle x\rangle \bigr) \\ &\quad{}\times G_{j_{1}}\bigl(\langle x\rangle\bigr)\cdots G_{j_{s}}\bigl(\langle x\rangle\bigr)\quad (m \geq r+s , \mbox{if } r > 0 \mbox{ or } m > s , \mbox{if }r =0). \end{aligned} \end{aligned}$$

Here the sums for (1) and (2) run over all nonnegative integers $c_{1}, \ldots, c_{r}$ and positive integers $j_{1},\ldots, j_{s}$ with $c_{1} + \cdots+ c_{r} + j_{1} + \cdots+ j_{s} = m$, and the sum for (3) runs over all positive integers $c_{1}, \ldots, c_{r},j_{1},\ldots, j_{s}$ with $c_{1} + \cdots+ c_{r} + j_{1} + \cdots+ j_{s} = m$.

For elementary facts about Fourier analysis, the reader may refer to any textbook (for example, see [11–13]).

As to $\alpha_{m}(\langle x\rangle)$, we note that the polynomial identity (1.7) follows immediately from Theorems 2.1 and 2.2, which is in turn derived from the Fourier series expansion of $\alpha_{m}(\langle x\rangle)$. We have

1.7 $$ \begin{aligned}[b] & \sum_{c_{1} + \cdots+ c_{r} + j_{1} + \cdots+ j_{s} = m} B_{c_{1}}(x)\cdots B_{c_{r}}(x)G_{j_{1}}(x)\cdots G_{j_{s}}(x) \\ &\quad =\frac{1}{m+r+s} \sum_{j=0}^{m} \binom{m+r+s}{j} \Delta _{m-j+1} B_{j}(x), \end{aligned} $$

where, for $l>s$,

1.8 $$ \begin{aligned}[b] \Delta_{l} & = \sum _{0 \leq a \leq r , 0 \leq c \leq s, r+s-l \leq a \leq r} \binom{r}{a} \binom{s}{c} (-1)^{c} 2^{s-c} \\ & \quad {}\times\sum_{c_{1} + \cdots+ c_{a} + j_{1} + \cdots+ j_{c} = l+a+c-r-s} B_{c_{1}}B_{c_{2}} \cdots B_{c_{a}}G_{j_{1}}\cdots G_{j_{c}} \\ & \quad {} - \sum_{c_{1} + \cdots+ c_{r} + j_{1} + \cdots+ j_{s} = l} B_{c_{1}}\cdots B_{c_{r}}G_{j_{1}}\cdots G_{j_{s}}. \end{aligned} $$

The obvious polynomial identities can be derived also for $\beta _{m}(\langle x\rangle)$ from Theorems 3.1 and 3.2. It is noteworthy that from the Fourier series expansion of the function $\sum_{k=1}^{m-1} \frac{1}{k(m-k)}B_{k}(\langle x\rangle )B_{m-k}(\langle x\rangle )$ we can derive the Faber-Pandharipande-Zagier identity (see [14–18]) and the Miki identity (see [16–19]). The reader may refer to the recent papers [20–24] for related results.

## The function $\alpha_{m}(\langle x\rangle)$

Let $\alpha_{m}(x) = \sum_{c_{1} + \cdots+ c_{r} + j_{1} + \cdots+ j_{s} = m} B_{c_{1}}(x)\cdots B_{c_{r}}(x)G_{j_{1}}(x)\cdots G_{j_{s}}(x)$ ($m > s$), where the sum is over all nonnegative integers $c_{1} , \ldots, c_{r}$ and positive integers $j_{1},\ldots, j_{s}$ satisfying $c_{1} + \cdots+ c_{r} + j_{1} + \cdots+ j_{s} = m$. Then we will consider the function

2.1 $$ \begin{aligned}[b] &\alpha_{m}\bigl(\langle x \rangle\bigr) \\ &\quad =\sum_{c_{1} + \cdots+ c_{r} + j_{1} + \cdots+ j_{s} = m} B_{c_{1}}\bigl(\langle x\rangle \bigr)\cdots B_{c_{r}}\bigl(\langle x\rangle\bigr)G_{j_{1}}\bigl( \langle x\rangle \bigr)\cdots G_{j_{s}}\bigl(\langle x\rangle\bigr), \end{aligned} $$

defined on $\mathbb{R}$, which is periodic with period 1.

The Fourier series of $\alpha_{m}(\langle x\rangle) $ is

2.2 $$ \sum_{n= -\infty}^{\infty} A_{n}^{(m)} e^{2\pi i nx} , $$

where

2.3 $$ A_{n}^{(m)}= \int_{0} ^{1} \alpha_{m}\bigl(\langle x \rangle\bigr) e^{-2\pi i nx} \,dx= \int_{0} ^{1} \alpha_{m}(x) e^{-2\pi i nx} \,dx. $$

To proceed, we need to observe the following. We have

2.4 $$\begin{aligned} \alpha_{m}'(x) & = \sum_{c_{1} + \cdots+ c_{r} + j_{1} + \cdots+ j_{s} = m , c_{1} \geq1} c_{1} B_{c_{1}-1}(x)B_{c_{2}}(x) \cdots B_{c_{r}}(x)G_{j_{1}}(x)\cdots G_{j_{s}}(x) \\ &\quad {} + \cdots + \sum_{c_{1} + \cdots+ c_{r} + j_{1} + \cdots+ j_{s} = m , c_{r} \geq1} B_{c_{1}}(x)\cdots B_{c_{r-1}} c_{r}B_{c_{r}-1}(x)G_{j_{1}}(x)\cdots G_{j_{s}}(x) \\ &\quad {} + \sum_{c_{1} + \cdots+ c_{r} + j_{1} + \cdots+ j_{s} = m , j_{1} \geq2} B_{c_{1}}(x)\cdots B_{c_{r}}(x)j_{1}G_{j_{1}-1}(x)G_{j_{2}}(x)\cdots G_{j_{s}}(x) \\ &\quad {} + \cdots + \sum_{c_{1} + \cdots+ c_{r} + j_{1} + \cdots+ j_{s} = m , j_{s} \geq2} B_{c_{1}}(x)\cdots B_{c_{r}}(x)G_{j_{1}}(x)\cdots G_{j_{s-1}}j_{s} G_{j_{s}-1}(x) \\ & = \sum_{c_{1} + \cdots+ c_{r} + j_{1} + \cdots+ j_{s} = m-1} (c_{1}+1) B_{c_{1}}(x)\cdots B_{c_{r}}(x)G_{j_{1}}(x)\cdots G_{j_{s}}(x) \\ & \quad {}+ \cdots + \sum_{c_{1} + \cdots+ c_{r} + j_{1} + \cdots+ j_{s} = m-1} (c_{r}+1)B_{c_{1}}(x) \cdots B_{c_{r}}(x)G_{j_{1}}(x)\cdots G_{j_{s}}(x) \\ &\quad {} + \sum_{c_{1} + \cdots+ c_{r} + j_{1} + \cdots+ j_{s} = m-1} (j_{1}+1)B_{c_{1}}(x) \cdots B_{c_{r}}(x)G_{j_{1}}(x)\cdots G_{j_{s}}(x) \\ &\quad {} + \cdots + \sum_{c_{1} + \cdots+ c_{r} + j_{1} + \cdots+ j_{s} = m-1}(j_{s}+1) B_{c_{1}}(x)\cdots B_{c_{r}}(x)G_{j_{1}}(x)\cdots G_{j_{s}}(x) \\ & = (m+r+s-1) \alpha_{m-1}(x). \end{aligned}$$

From this, we obtain

2.5 $$ \biggl(\frac{\alpha_{m+1}(x)}{m+r+s} \biggr)' = \alpha_{m}(x) $$

and

2.6 $$ \int_{0} ^{1} \alpha_{m}(x) \,dx = \frac{1}{m+r+s} \bigl(\alpha_{m+1}(1) - \alpha_{m+1} (0) \bigr). $$

For $m > s$, we set

2.7 $$ \begin{aligned}[b] \Delta_{m} &= \alpha_{m}(1) - \alpha_{m } (0) \\ & = \sum_{c_{1} + \cdots+ c_{r} + j_{1} + \cdots+ j_{s} = m} \bigl( B_{c_{1}}(1)\cdots B_{c_{r}}(1)G_{j_{1}}(1)\cdots G_{j_{s}}(1) - B_{c_{1}} \cdots B_{c_{r}}G_{j_{1}}\cdots G_{j_{s}} \bigr) \\ & = \sum_{c_{1} + \cdots+ c_{r} + j_{1} + \cdots+ j_{s} = m} (B_{c_{1}} + \delta_{1,c_{1}}) \cdots(B_{c_{r}} + \delta_{1,c_{r}}) (- G_{j_{1}} + 2\delta_{1,j_{1}}) \cdots (- G_{j_{s}} + 2 \delta_{1,j_{s}}) \\ &\quad {} - \sum_{c_{1} + \cdots+ c_{r} + j_{1} + \cdots+ j_{s} = m} B_{c_{1}} \cdots B_{c_{r}}G_{j_{1}}\cdots G_{j_{s}} \\ & = \sum_{ 0 \leq a \leq r, 0 \leq c \leq s , r+s-m \leq a \leq r} \binom{r}{a} \binom{s}{c}(-1)^{c} 2^{s-c} \\ &\quad {}\times \sum_{c_{1} + \cdots+ c_{a} + j_{1} + \cdots+ j_{c} = m+a+c-r-s} B_{c_{1}}\cdots B_{c_{a}}G_{j_{1}}\cdots G_{j_{c}} \\ &\quad {} - \sum_{c_{1} + \cdots+ c_{r} + j_{1} + \cdots+ j_{s} = m} B_{c_{1}}\cdots B_{c_{r}}G_{j_{1}}\cdots G_{j_{s}}. \end{aligned} $$

Note here that the sum over all $c_{1} + \cdots+ c_{r} + j_{1} + \cdots+ j_{s} = m$ of any term with *a* of $B_{c_{e}}$, $r-a$ of $\delta_{1,c_{f}}$ ($1 \leq e,f \leq r$), *c* of $-G_{j_{u}}$, and $s-c$ of $2\delta _{1,j_{v}}$ ($1 \leq u,v \leq s$) all give the same sum

2.8 $$ \begin{aligned}[b] & \sum_{c_{1} + \cdots+ c_{r} + j_{1} + \cdots+ j_{s} = m}B_{c_{1}} \cdots B_{c_{a}} \delta_{1,c_{a+1}} \cdots \delta_{1,c_{r}}(-G_{j_{1}}) \cdots (-G_{j_{c}}) \\ &\qquad {}\times(2\delta_{1,j_{c+1}})\cdots(2\delta_{1,j_{s}}) \\ &\quad = \sum_{c_{1} + \cdots+ c_{a} + j_{1} + \cdots+ j_{c} = m+a+c-r-s}(-1)^{c} 2^{s-c} B_{c_{1}} \cdots B_{c_{a}}G_{j_{1}}\cdots G_{j_{c}}, \end{aligned} $$

which is not an empty sum as long as $m + a + c - r -s \geq c $.

We now see that

2.9 $$ \alpha_{m}(0) = \alpha_{m}(1) \quad \Longleftrightarrow\quad \Delta_{m} = 0 $$

and

2.10 $$ \int_{0} ^{1} \alpha_{m}(x) \,dx = \frac{1}{m+r+s} \Delta_{m+1}. $$

We are now going to determine the Fourier coefficients $A_{n}^{(m)}$.

*Case 1*: $n \neq0$. We have

2.11 $$ \begin{aligned}[b] A_{n}^{(m)} & = \int_{0} ^{1} \alpha_{m} (x) e^{-2\pi i nx} \,dx \\ & = - \frac{1}{2\pi i n} \bigl[\alpha_{m} (x) e^{-2\pi i nx} \bigr]_{0}^{1} + \frac{1}{2\pi i n} \int_{0} ^{1} \alpha_{m}' (x) e^{-2\pi i nx} \, dx \\ & = - \frac{1}{2\pi i n} \bigl(\alpha_{m} (1) - \alpha_{m} (0) \bigr) + \frac{m+r+s-1}{2\pi i n} \int_{0} ^{1} \alpha_{m-1}(x) e^{-2\pi i nx} \,dx \\ & = \frac{m+r+s-1}{2\pi i n} A_{n}^{(m-1)} - \frac{1}{2 \pi i n} \Delta _{m} \\ & = \frac{m+r+s-1}{2\pi i n} \biggl( \frac{m+r+s-2}{2\pi i n} A_{n}^{(m-2)} - \frac{1}{2\pi i n} \Delta_{m-1} \biggr) - \frac {1}{2\pi i n} \Delta_{m} \\ & = \frac{(m+r+s-1)_{2}}{(2\pi i n)^{2}} A_{n}^{(m-2)} - \sum _{j=1}^{2} \frac{(m+r+s-1)_{j-1}}{(2 \pi i n)^{j}} \Delta_{m-j+1} \\ &=\cdots \\ & = \frac{(m+r+s-1)_{m-s}}{(2\pi i n)^{m-s}} A_{n}^{(s)} - \sum _{j=1}^{m-s} \frac{(m+r+s-1)_{j-1}}{(2\pi i n)^{j}} \Delta_{m-j+1} \\ & = - \sum_{j=1}^{m-s} \frac{(m+r+s-1)_{j-1}}{(2\pi i n)^{j}} \Delta_{m-j+1}. \end{aligned} $$

Thus we have shown that

2.12 $$ A_{n}^{(m)} = - \frac{1}{m+r+s} \sum _{j=1}^{m-s} \frac {(m+r+s)_{j}}{(2\pi i n)^{j}} \Delta_{m-j+1}. $$

*Case 2*: $n = 0 $. We have

2.13 $$ A_{0}^{(m)} = \int_{0} ^{1} \alpha_{m} (x) \,dx = \frac{1}{m+r+s} \Delta_{m+1}. $$

$\alpha_{m}(\langle x\rangle)$ ($m>s$) is piecewise $C^{\infty}$. Moreover, $\alpha_{m}(\langle x\rangle) $ is continuous for those integers $m>s$ with $\Delta_{m} = 0$, and discontinuous with jump discontinuities at integers for those integers $m>s$ with $\Delta_{m} \neq0$.

Assume first that $\Delta_{m} = 0$, for an integer $m>s$. Then $\alpha _{m} (0) = \alpha_{m} (1)$.

Hence $\alpha_{m} (\langle x\rangle)$ is piecewise $C^{\infty}$, and continuous. Thus the Fourier series of $\alpha_{m} (\langle x\rangle)$ converges uniformly to $\alpha_{m} (\langle x\rangle)$, and

2.14 $$ \begin{aligned}[b] \alpha_{m} \bigl(\langle x \rangle\bigr) & = \frac{1}{m+r+s} \Delta_{m+1} \\ &\quad {} + \sum_{n= - \infty, n \neq0}^{\infty} \Biggl( - \frac{1}{m+r+s} \sum_{j=1}^{m-s} \frac{(m+r+s)_{j}}{(2\pi i n)^{j}} \Delta_{m-j+1} \Biggr)e^{2\pi i nx} \\ & = \frac{1}{m+r+s} \Delta_{m+1} + \frac{1}{m+r+s} \sum _{j=1}^{m-s} \binom{m+r+s}{j} \Delta_{m-j+1} \\ &\quad {} \times \Biggl( -j! \sum_{n= - \infty, n \neq0}^{\infty} \frac {e^{2\pi i nx}}{(2\pi i n)^{j}} \Biggr) \\ & = \frac{1}{m+r+s} \Delta_{m+1}+ \frac{1}{m+r+s} \sum _{j=2}^{m-s} \binom{m+r+s}{j} \Delta_{m-j+1} B_{j}\bigl(\langle x\rangle\bigr) \\ &\quad {} + \Delta_{m} \times \textstyle\begin{cases} B_{1}(\langle x\rangle),& \textrm{for } x\notin\mathbb{Z},\\ 0,&\textrm{for }x\in\mathbb{Z}. \end{cases}\displaystyle \end{aligned} $$

We are now ready to state our first result.

### Theorem 2.1

*For each integer* $l > s$, *we let*

$$ \begin{aligned}[b] \Delta_{l} & = \sum _{0 \leq a \leq r , 0 \leq c \leq s,r+s-l \leq a \leq r} \binom{r}{a} \binom{s}{c} (-1)^{c} 2^{s-c} \\ &\quad {}\times\sum_{c_{1} + \cdots+ c_{a} + j_{1} + \cdots+ j_{c} = l+a+c-r-s} B_{c_{1}}\cdots B_{c_{a}}G_{j_{1}}\cdots G_{j_{c}} \\ &\quad {} - \sum_{c_{1} + \cdots+ c_{r} + j_{1} + \cdots+ j_{s} = l} B_{c_{1}}\cdots B_{c_{r}}G_{j_{1}}\cdots G_{j_{s}}. \end{aligned} $$

*Assume that* $\Delta_{m} = 0$, *for an integer* $m > s$. *Then we have the following*:

- $\sum_{c_{1} + \cdots+ c_{r} + j_{1} + \cdots+ j_{s} = m} B_{c_{1}}(\langle x\rangle)\cdots B_{c_{r}}(\langle x\rangle )G_{j_{1}}(\langle x\rangle)\cdots G_{j_{s}}(\langle x\rangle)$ *has the Fourier series expansion*
  $$ \begin{aligned}[b] & \sum_{c_{1} + \cdots+ c_{r} + j_{1} + \cdots+ j_{s} = m} B_{c_{1}}\bigl(\langle x\rangle\bigr)\cdots B_{c_{r}}\bigl(\langle x\rangle\bigr)G_{j_{1}}\bigl(\langle x\rangle \bigr)\cdots G_{j_{s}}\bigl(\langle x\rangle\bigr) \\ &\quad = \frac{1}{m+r+s} \Delta_{m+1} + \sum _{n= - \infty, n \neq0}^{\infty} \Biggl( -\frac{1}{m+r+s} \sum _{j=1}^{m-s} \frac{(m+r+s)_{j}}{(2\pi i n)^{j}} \Delta_{m-j+1} \Biggr)e^{2\pi i nx}, \end{aligned} $$
  *for all* $x \in\mathbb{R}$, *where the convergence is uniform*.
- $$ \begin{aligned}[b] & \sum_{c_{1} + \cdots+ c_{r} + j_{1} + \cdots+ j_{s} = m} B_{c_{1}}\bigl(\langle x\rangle\bigr)\cdots B_{c_{r}}\bigl(\langle x\rangle\bigr)G_{j_{1}}\bigl(\langle x\rangle \bigr)\cdots G_{j_{s}}\bigl(\langle x\rangle\bigr) \\ &\quad = \frac{1}{m+r+s} \Delta_{m+1}+ \frac{1}{m+r+s} \sum _{j=2}^{m-s} \binom{m+r+s}{j} \Delta_{m-j+1} B_{j}\bigl(\langle x\rangle\bigr) , \end{aligned} $$
  *for all* $x \in\mathbb{R}$, *where* $B_{j}(\langle x\rangle)$ *is the Bernoulli function*.

Assume next that $\Delta_{m} \neq0$, for an integer $m>s$. Then $\alpha_{m}(0) \neq\alpha_{m}(1)$. Hence $\alpha_{m} (\langle x\rangle)$ is piecewise $C^{\infty}$, and discontinuous with jump discontinuities at integers. The Fourier series of $\alpha_{m} (\langle x\rangle)$ converges pointwise to $\alpha_{m} (\langle x\rangle)$, for $x \notin\mathbb {Z}$, and converges to

2.15 $$ \frac{1}{2} \bigl(\alpha_{m} (0)+ \alpha_{m} (1) \bigr) = \alpha_{m} (0) + \frac{1}{2} \Delta_{m} , $$

for $x \in\mathbb{Z}$.

Now, we are ready to state our second result.

### Theorem 2.2

*For each integer* $l > s$, *we let*

$$ \begin{aligned}[b] \Delta_{l} & = \sum _{0 \leq a \leq r , 0 \leq c \leq s, r+s-l \leq a \leq r} \binom{r}{a} \binom{s}{c} (-1)^{c} 2^{s-c} \\ & \quad {}\times\sum_{c_{1} + \cdots+ c_{a} + j_{1} + \cdots+ j_{c} = l+a+c-r-s} B_{c_{1}}B_{c_{2}} \cdots B_{c_{a}}G_{j_{1}}\cdots G_{j_{c}} \\ &\quad {} - \sum_{c_{1} + \cdots+ c_{r} + j_{1} + \cdots+ j_{s} = l} B_{c_{1}}\cdots B_{c_{r}}G_{j_{1}}\cdots G_{j_{s}}. \end{aligned} $$

*Assume that* $\Delta_{m} \neq0$, *for an integer* $m > s$. *Then we have the following*:

- $$\begin{aligned} & \frac{1}{m+r+s} \Delta_{m+1} \\ &\qquad {} + \sum_{n= - \infty, n \neq0}^{\infty} \Biggl( - \frac{1}{m+r+s} \sum_{j=1}^{m-s} \frac{(m+r+s)_{j}}{(2\pi i n)^{j}} \Delta_{m-j+1} \Biggr)e^{2\pi i nx} \\ & \quad = \textstyle\begin{cases} \sum_{c_{1} + \cdots+ c_{r} + j_{1} + \cdots+ j_{s} = m} B_{c_{1}}(\langle x\rangle)\cdots B_{c_{r}}(\langle x\rangle)\\ \quad {}\times G_{j_{1}}(\langle x\rangle)\cdots G_{j_{s}}(\langle x\rangle) ,& \textit{for } x\notin\mathbb{Z},\\ \sum_{c_{1} + \cdots+ c_{r} + j_{1} + \cdots+ j_{s} = m} B_{c_{1}} \cdots B_{c_{r}}G_{j_{1}} \cdots G_{j_{s}}+ \frac{1}{2}\Delta_{m} , & \textit{for } x\in\mathbb{Z}. \end{cases}\displaystyle \end{aligned} $$
- $$\begin{aligned}& \begin{gathered} \frac{1}{m+r+s} \sum _{j=0}^{m} \binom{m+r+s}{j} \Delta _{m-j+1} B_{j}\bigl(\langle x\rangle\bigr) \\ \quad = \sum_{c_{1} + \cdots+ c_{r} + j_{1} + \cdots+ j_{s} = m} B_{c_{1}}\bigl(\langle x \rangle\bigr)\cdots B_{c_{r}}\bigl(\langle x\rangle\bigr)G_{j_{1}} \bigl(\langle x\rangle \bigr)\cdots G_{j_{s}}\bigl(\langle x\rangle\bigr) , \quad \textit{for } x \notin\mathbb{Z}, \end{gathered} \\& \begin{gathered} \frac{1}{m+r+s} \sum _{j=0, j\neq1}^{m} \binom{m+r+s}{j} \Delta _{m-j+1} B_{j}\bigl(\langle x\rangle\bigr) \\ \quad = \sum_{c_{1} + \cdots+ c_{r} + j_{1} + \cdots+ j_{s} = m} B_{c_{1}} \cdots B_{c_{r}}G_{j_{1}} \cdots G_{j_{s}}+ \frac{1}{2} \Delta_{m}, \quad \textit {for } x \in\mathbb{Z}. \end{gathered} \end{aligned}$$

## The function $\beta_{m}(\langle x\rangle)$

Let $\beta_{m}(x)$ = $\sum_{c_{1} + \cdots+ c_{r} + j_{1} + \cdots+ j_{s} = m} \frac{1}{c_{1}! \cdots c_{r}! j_{1}! \cdots j_{s}!} B_{c_{1}}(x) \cdots B_{c_{r}}(x) G_{j_{1}}(x) \cdots G_{j_{s}}(x) $ ($m > s$), where the sum is over all nonnegative integers $c_{1}, \ldots , c_{r}$ and positive integers $j_{1},\ldots, j_{s}$ satisfying $c_{1} + \cdots+ c_{r} + j_{1} + \cdots+ j_{s} = m$. Then we consider the function

$$\begin{aligned} \beta_{m}\bigl(\langle x\rangle\bigr) &= \sum _{c_{1} + \cdots+ c_{r} + j_{1} + \cdots + j_{s} = m} \frac{1}{c_{1}! \cdots c_{r}! j_{1}! \cdots j_{s}!} B_{c_{1}}\bigl(\langle x \rangle\bigr)\cdots B_{c_{r}}\bigl(\langle x\rangle\bigr)\\ &\quad {} \times G_{j_{1}}\bigl(\langle x\rangle\bigr)\cdots G_{j_{s}}\bigl(\langle x\rangle\bigr), \end{aligned} $$

defined on $\mathbb{R}$, which is periodic with period 1.

The Fourier series of $\beta_{m}(\langle x\rangle)$ is

3.1 $$ \sum_{n=-\infty}^{\infty}B_{n}^{(m)}e^{2\pi i n x}, $$

where

3.2 $$ B_{n}^{(m)} = \int_{0}^{1} \beta_{m}\bigl(\langle x \rangle\bigr)e^{-2\pi i n x}\,dx = \int_{0}^{1} \beta_{m}(x)e^{-2\pi i n x} \,dx. $$

To continue further, we need to observe the following:

3.3 $$\begin{aligned} &\beta'_{m}(x) \\ &\quad = \sum_{c_{1} + \cdots+ c_{r} + j_{1} + \cdots+ j_{s} = m , c_{1} \geq1} \frac{1}{(c_{1}-1)!c_{2}! \cdots c_{r}! j_{1}! \cdots j_{s}!} B_{c_{1}-1}(x)B_{c_{2}}(x)\cdots B_{c_{r}}(x) \\ &\qquad {}\times G_{j_{1}}(x)\cdots G_{j_{s}}(x) \\ &\qquad {} + \cdots \\ &\qquad {}+ \sum_{c_{1} + \cdots+ c_{r} + j_{1} + \cdots+ j_{s} = m , c_{r} \geq1} \frac{1}{c_{1}! \cdots c_{r-1}! (c_{r} -1)! j_{1}! \cdots j_{s}!}B_{c_{1}}(x) \cdots B_{c_{r-1}}(x) B_{c_{r}-1}(x) \\ &\qquad {}\times G_{j_{1}}(x)\cdots G_{j_{s}}(x) \\ &\qquad {} + \sum_{c_{1} + \cdots+ c_{r} + j_{1} + \cdots+ j_{s} = m , j_{1} \geq2} \frac{1}{c_{1}! \cdots c_{r}! (j_{1}-1)!j_{2}! \cdots j_{s}!}B_{c_{1}}(x) \cdots B_{c_{r}}(x) \\ &\qquad {}\times G_{j_{1}-1}(x)G_{j_{2}}(x)\cdots G_{j_{s}}(x) \\ &\qquad {} + \cdots \\ &\qquad {}+ \sum_{c_{1} + \cdots+ c_{r} + j_{1} + \cdots+ j_{s} = m , j_{s} \geq2} \frac{1}{c_{1}! \cdots c_{r}! j_{1}! \cdots j_{s-1}! (j_{s}-1)!} B_{c_{1}}(x)\cdots B_{c_{r}}(x) \\ &\qquad {}\times G_{j_{1}}(x)\cdots G_{j_{s-1}}(x) G_{j_{s}-1}(x) \\ &\quad = \sum _{c_{1} + \cdots+ c_{r} + j_{1} + \cdots+ j_{s} = m-1} \frac{1}{c_{1}! \cdots c_{r}! j_{1}! \cdots j_{s}!} B_{c_{1}}(x)\cdots B_{c_{r}}(x)G_{j_{1}}(x)\cdots G_{j_{s}}(x) \\ &\qquad {} + \cdots+ \sum_{c_{1} + \cdots+ c_{r} + j_{1} + \cdots+ j_{s} = m-1} \frac{1}{c_{1}! \cdots c_{r}! j_{1}! \cdots j_{s}!} B_{c_{1}}(x)\cdots B_{c_{r}}(x) G_{j_{1}}(x)\cdots G_{j_{s}}(x) \\ &\qquad {} + \sum_{c_{1} + \cdots+ c_{r} + j_{1} + \cdots+ j_{s} = m-1} \frac {1}{c_{1}! \cdots c_{r}! j_{1}! \cdots j_{s}!}B_{c_{1}}(x) \cdots B_{c_{r}}(x) G_{j_{1}}(x)\cdots G_{j_{s}}(x) \\ &\qquad {} + \cdots + \sum_{c_{1} + \cdots+ c_{r} + j_{1} + \cdots+ j_{s} = m-1} \frac{1}{c_{1}! \cdots c_{r}! j_{1}! \cdots j_{s}!} B_{c_{1}}(x)\cdots B_{c_{r}}(x)G_{j_{1}}(x)\cdots G_{j_{s}}(x) \\ &\quad =(r+s)\beta_{m-1}(x). \end{aligned}$$

From this, we have

3.4 $$ \biggl(\frac{\beta_{m+1}(x)}{r+s} \biggr)'= \beta_{m}(x) $$

and

3.5 $$ \int_{0}^{1}\beta_{m}(x)\,dx= \frac{1}{r+s} \bigl(\beta_{m+1}(1)- \beta_{m+1}(0) \bigr). $$

For $m > s$, we put

3.6 $$\begin{aligned}& \begin{aligned}[b] \Omega_{m} &= \beta_{m}(1)-\beta_{m}(0) \\ &=\sum_{c_{1} + \cdots+ c_{r} + j_{1} + \cdots+ j_{s} = m} \frac{1}{c_{1}! \cdots c_{r}! j_{1}! \cdots j_{s}!} B_{c_{1}}(1) \cdots B_{c_{r}}(1) G_{j_{1}}(1) \cdots G_{j_{s}}(1) \\ &\quad {}- \sum_{c_{1} + \cdots+ c_{r} + j_{1} + \cdots+ j_{s} = m} \frac{1}{c_{1}! \cdots c_{r}! j_{1}! \cdots j_{s}!}B_{c_{1}} \cdots B_{c_{r}} G_{j_{1}} \cdots G_{j_{s}} \\ &=\sum_{c_{1} + \cdots+ c_{r} + j_{1} + \cdots+ j_{s} = m} \frac{1}{c_{1}! \cdots c_{r}! j_{1}! \cdots j_{s}!} (B_{c_{1}} + \delta_{1,c_{1}}) \cdots (B_{c_{r}} + \delta_{1,c_{r}}) \\ &\quad {}\times(- G_{j_{1}} + 2\delta_{1,j_{1}}) \cdots(- G_{j_{s}} + 2 \delta _{1,j_{s}}) \\ &\quad {} - \sum_{c_{1} + \cdots+ c_{r} + j_{1} + \cdots+ j_{s} = m} \frac{1}{c_{1}! \cdots c_{r}! j_{1}! \cdots j_{s}!} B_{c_{1}} \cdots B_{c_{r}} G_{j_{1}} \cdots G_{j_{s}} \\ & = \sum_{ 0 \leq a \leq r, 0 \leq c \leq s , r+s-m \leq a \leq r} \binom{r}{a} \binom{s}{c}(-1)^{c} 2^{s-c} \\ &\quad {} \times \sum_{c_{1} + \cdots+ c_{a} + j_{1} + \cdots+ j_{c} = m+a+c-r-s} \frac{1}{c_{1}! \cdots c_{a}! j_{1}! \cdots j_{c}!}B_{c_{1}} \cdots B_{c_{a}}G_{j_{1}}\cdots G_{j_{c}} \\ &\quad {} - \sum_{c_{1} + \cdots+ c_{r} + j_{1} + \cdots+ j_{s} = m} \frac{1}{c_{1}! \cdots c_{r}! j_{1}! \cdots j_{s}!} B_{c_{1}} \cdots B_{c_{r}}G_{j_{1}}\cdots G_{j_{s}}. \end{aligned} \end{aligned}$$

3.7 $$\begin{aligned}& \beta_{m}(0) = \beta_{m}(1)\quad \Leftrightarrow\quad \Omega_{m} = 0, \end{aligned}$$

and

3.8 $$ \int_{0}^{1}\beta_{m}(x)\,dx = \frac{1}{r+s} \Omega_{m+1}. $$

We now would like to determine the Fourier coefficients $B_{n}^{(m)}$.

*Case 1*: $n\neq0$. We have

3.9 $$ \begin{aligned}[b] B_{n}^{(m)}&= \int_{0}^{1}\beta_{m}(x)e^{-2\pi i n x} \,dx \\ &=-\frac{1}{2\pi i n} \bigl[\beta_{m}(x)e^{-2\pi i n x} \bigr]_{0}^{1} + \frac{1}{2\pi i n} \int_{0}^{1}\beta'_{m}(x)e^{-2\pi i n x} \,dx \\ &=-\frac{1}{2\pi i n} \bigl(\beta_{m}(1)-\beta_{m}(0) \bigr) + \frac {r+s}{2\pi i n} \int_{0}^{1}\beta_{m-1}(x)e^{-2\pi i n x} \,dx \\ &=\frac{r+s}{2\pi i n}B_{n}^{(m-1)}- \frac{1}{2\pi i n} \Omega_{m}, \end{aligned} $$

from which we can deduce that

3.10 $$ B_{n}^{(m)}= - \sum _{j=1}^{m-s} \frac{(r+s)^{j-1}}{(2\pi i n)^{j}} \Omega_{m-j+1}. $$

*Case 2*: $n=0$. We have

3.11 $$ B_{0}^{(m)}= \int_{0}^{1}\beta_{m}(x)\,dx= \frac{1}{r+s} \Omega_{m+1}. $$

$\beta_{m}(\langle x\rangle)$ ($m\geq s$) is piecewise $C^{\infty}$. Moreover, $\beta_{m}(\langle x\rangle)$ is continuous for those integers $m > s$ with $\Delta_{m} =0$, and discontinuous with jump discontinuities at integers for those integers $m > s$ with $\Delta_{m} \neq0$.

Assume first that $\Delta_{m} =0$, for an integer $m > s$. Then $\beta _{m}(0)=\beta_{m}(1)$. Hence $\beta_{m}(\langle x\rangle)$ is piecewise $C^{\infty}$, and continuous. Thus the Fourier series of $\beta_{m}(\langle x\rangle)$ converges uniformly to $\beta_{m}(\langle x\rangle)$, and

3.12 $$ \begin{aligned}[b] B_{n}^{(m)} & = \frac{1}{r+s} \Omega_{m+1} + \sum_{n=-\infty,n\neq0}^{\infty} \Biggl(- \sum_{j=1}^{m-s} \frac {(r+s)^{j-1}}{(2\pi i n)^{j}} \Omega_{m-j+1} \Biggr)e^{2\pi i n x} \\ &= \frac{1}{r+s} \Omega_{m+1} + \sum_{j=1}^{m-s} \frac {(r+s)^{j-1}}{j!}\Omega_{m-j+1} \times \Biggl(- j! \sum _{n=-\infty,n\neq0}^{\infty} \frac{e^{2\pi i n x}}{(2\pi i n)^{j}} \Biggr) \\ &=\frac{1}{r+s} \Omega_{m+1} + \sum_{j=2}^{m-s} \frac {(r+s)^{j-1}}{j!}\Omega_{m-j+1} B_{j}\bigl(\langle x\rangle \bigr) \\ & \quad {}+ \Omega_{m} \times \textstyle\begin{cases} B_{1}(\langle x\rangle),&\textrm{for } x\notin\mathbb {Z},\\ 0,&\textrm{for } x\in\mathbb{Z}. \end{cases}\displaystyle \end{aligned} $$

Now, we are ready to state our first result.

### Theorem 3.1

*For each integer* $l > s$, *we let*

$$ \begin{aligned}[b] \Omega_{l} &= \sum _{ 0 \leq a \leq r, 0 \leq c \leq s , r+s-l \leq a \leq r} \binom{r}{a} \binom{s}{c}(-1)^{c} 2^{s-c} \\ & \quad {}\times\sum_{c_{1} + \cdots+ c_{a} + j_{1} + \cdots+ j_{c} = l+a+c-r-s} \frac {1}{c_{1}! \cdots c_{a}! j_{1}! \cdots j_{c}!}B_{c_{1}} \cdots B_{c_{a}}G_{j_{1}}\cdots G_{j_{c}} \\ &\quad {} - \sum_{c_{1} + \cdots+ c_{r} + j_{1} + \cdots+ j_{s} = l} \frac{1}{c_{1}! \cdots c_{r}! j_{1}! \cdots j_{s}!} B_{c_{1}} \cdots B_{c_{r}}G_{j_{1}}\cdots G_{j_{s}}. \end{aligned} $$

*Assume that* $\Omega_{m} = 0$, *for an integer* $m > s$. *Then we have the following*:

- $\sum_{c_{1} + \cdots+ c_{r} + j_{1} + \cdots+ j_{s} = m} \frac {1}{c_{1}! \cdots c_{r}! j_{1}! \cdots j_{s}!} B_{c_{1}}(\langle x\rangle)\cdots B_{c_{r}}(\langle x\rangle) \times G_{j_{1}}(\langle x\rangle)\cdots G_{j_{s}}(\langle x\rangle)$ *has the Fourier series expansion*
  $$ \begin{aligned}[b] &\sum_{c_{1} + \cdots+ c_{r} + j_{1} + \cdots+ j_{s} = m} \frac{1}{c_{1}! \cdots c_{r}! j_{1}! \cdots j_{s}!} B_{c_{1}}\bigl(\langle x\rangle\bigr)\cdots B_{c_{r}}\bigl(\langle x\rangle\bigr) \\ &\qquad {}\times G_{j_{1}}\bigl(\langle x\rangle\bigr)\cdots G_{j_{s}} \bigl(\langle x\rangle\bigr) \\ &\quad = \frac{1}{r+s} \Omega_{m+1} + \sum _{n=-\infty,n\neq0}^{\infty} \Biggl(- \sum _{j=1}^{m-s} \frac {(r+s)^{j-1}}{(2\pi i n)^{j}}\Omega_{m-j+1} \Biggr)e^{2\pi i n x}, \end{aligned} $$
  *for all* $x \in\mathbb{R}$, *where the convergence is uniform*.
- $$ \begin{aligned}[b] &\sum_{c_{1} + \cdots+ c_{r} + j_{1} + \cdots+ j_{s} = m} \frac {1}{c_{1}! \cdots c_{r}! j_{1}! \cdots j_{s}!} B_{c_{1}}\bigl(\langle x\rangle\bigr)\cdots B_{c_{r}}\bigl(\langle x\rangle\bigr) \\ &\qquad {}\times G_{j_{1}}\bigl(\langle x\rangle\bigr)\cdots G_{j_{s}} \bigl(\langle x\rangle\bigr) \\ & \quad = \sum_{j=0, j \neq1}^{m-s} \frac{(r+s)^{j-1}}{j!} \Omega_{m-j+1} B_{j}\bigl(\langle x\rangle\bigr), \end{aligned} $$
  *for all* $x \in\mathbb{R}$, *where* $B_{j}(\langle x\rangle)$ *is the Bernoulli function*.

Assume next that $\Omega_{m} \neq0$, for an integer $m > s$. Then $\beta_{m}(0)\neq\beta_{m}(1)$. Hence $\beta_{m}(\langle x\rangle )$ is piecewise $C^{\infty}$ and discontinuous with jump discontinuities at integers. The Fourier series of $\beta_{m}(\langle x\rangle)$ converges pointwise to $\beta_{m}(\langle x\rangle)$, for $x \notin\mathbb{Z}$, and converges to

3.13 $$ \frac{1}{2}\bigl(\beta_{m}(0)+ \beta_{m}(1)\bigr)=\beta_{m}(0) + \frac{1}{2} \Omega_{m}, $$

for $x\in\mathbb{Z}$.

We are now ready to state our second result.

### Theorem 3.2

*For each integer* $l > s$, *we let*

$$ \begin{aligned}[b] \Omega_{l} &= \sum _{ 0 \leq a \leq r, 0 \leq c \leq s , r+s-l \leq a \leq r} \binom{r}{a} \binom{s}{c}(-1)^{c} 2^{s-c} \\ &\quad {}\times \sum_{c_{1} + \cdots+ c_{a} + j_{1} + \cdots+ j_{c} = l+a+c-r-s} \frac{1}{c_{1}! \cdots c_{a}! j_{1}! \cdots j_{c}!}B_{c_{1}} \cdots B_{c_{a}}G_{j_{1}}\cdots G_{j_{c}} \\ &\quad {} - \sum_{c_{1} + \cdots+ c_{r} + j_{1} + \cdots+ j_{s} = l} \frac{1}{c_{1}! \cdots c_{r}! j_{1}! \cdots j_{s}!} B_{c_{1}} \cdots B_{c_{r}}G_{j_{1}}\cdots G_{j_{s}}. \end{aligned} $$

*Assume that* $\Omega_{m}\neq0$, *for an integer* $m > s$. *Then we have the following*:

- $$ \begin{aligned}[b] &\frac{1}{r+s} \Omega_{m+1} + \sum_{n=-\infty,n\neq0}^{\infty} \Biggl(-\sum _{j=1}^{m-s} \frac{(r+s)^{j-1}}{(2\pi i n)^{j}}\Omega _{m-j+1} \Biggr)e^{2\pi i n x} \\ &\quad = \textstyle\begin{cases} \sum_{c_{1} + \cdots+ c_{r} + j_{1} + \cdots+ j_{s} = m} \frac{1}{c_{1}! \cdots c_{r}! j_{1}! \cdots j_{s}!} B_{c_{1}}(\langle x\rangle)\cdots B_{c_{r}}(\langle x\rangle)\\ \quad {}\times G_{j_{1}}(\langle x\rangle)\cdots G_{j_{s}}(\langle x\rangle ),&\textit{for } x\notin\mathbb{Z},\\ \sum_{c_{1} + \cdots+ c_{r} + j_{1} + \cdots+ j_{s} = m} \frac{1}{c_{1}! \cdots c_{r}! j_{1}! \cdots j_{s}!} B_{c_{1}}B_{c_{2}}\cdots B_{c_{r}}G_{j_{1}} \cdots G_{j_{s}} + \frac{1}{2}\Omega_{m} ,& \textit{for } x\in\mathbb{Z}. \end{cases}\displaystyle \end{aligned} $$
- $$ \begin{aligned}[b] & \sum_{j=0}^{m-s} \frac{(r+s)^{j-1}}{j!}\Omega_{m-j+1} B_{j}\bigl(\langle x\rangle \bigr) \\ &\quad = \sum_{c_{1} + \cdots+ c_{r} + j_{1} + \cdots+ j_{s} = m} \frac{1}{c_{1}! \cdots c_{r}! j_{1}! \cdots j_{s}!} B_{c_{1}} \bigl(\langle x\rangle\bigr)\cdots B_{c_{r}}\bigl(\langle x\rangle\bigr) \\ &\qquad {}\times G_{j_{1}}\bigl(\langle x\rangle\bigr)\cdots G_{j_{s}} \bigl(\langle x\rangle \bigr),\quad\textrm{for } x\notin\mathbb{Z}, \\ & \sum_{j=0,j\neq1}^{m-s} \frac{(r+s)^{j-1}}{j!} \Omega_{m-j+1} B_{j}\bigl(\langle x\rangle\bigr) \\ &\quad = \sum_{c_{1} + \cdots+ c_{r} + j_{1} + \cdots+ j_{s} = m} \frac{1}{c_{1}! \cdots c_{r}! j_{1}! \cdots j_{s}!} B_{c_{1}}B_{c_{2}}\cdots B_{c_{r}}G_{j_{1}} \cdots G_{j_{s}} \\ & \qquad {}+ \frac{1}{2}\Omega_{m},\quad\textrm{for }x\in\mathbb{Z}. \end{aligned} $$

## The function $\gamma_{m}(\langle x\rangle)$

Here we assume that *r*, *s* and *m* satisfy $m \geq r+s$ if $r > 0$ or $m > s$ if $r = 0$.

Let $\gamma_{r,s,m}(x)=\sum_{c_{1} + \cdots+ c_{r} + j_{1} + \cdots+ j_{s} = m} \frac{1}{c_{1} \cdots c_{r} j_{1} \cdots j_{s}} B_{c_{1}}(x)\cdots B_{c_{r}}(x)G_{j_{1}}(x)\cdots G_{j_{s}}(x)$, where the sum is over all positive integers $c_{1}, \ldots, c_{r}, j_{1}, \ldots, j_{s}$ satisfying $c_{1} + \cdots+ c_{r} + j_{1} + \cdots+ j_{s} = m$. Then we will consider the function

4.1 $$ \begin{aligned}[b] \gamma_{r,s,m}(x) &=\sum _{c_{1} + \cdots+ c_{r} + j_{1} + \cdots+ j_{s} = m} \frac{1}{c_{1} \cdots c_{r} j_{1} \cdots j_{s}} B_{c_{1}}\bigl( \langle x\rangle \bigr)\cdots B_{c_{r}}\bigl(\langle x\rangle\bigr) \\ &\quad {}\times G_{j_{1}}\bigl(\langle x\rangle\bigr)\cdots G_{j_{s}} \bigl(\langle x\rangle\bigr), \end{aligned} $$

defined on $\mathbb{R}$, which is periodic with period 1.

The Fourier series of $\gamma_{r,s,m}(\langle x\rangle)$ is

4.2 $$ \sum_{n=-\infty}^{\infty}C_{n}^{(r,s,m)}e^{2\pi i n x}, $$

where

4.3 $$ C_{n}^{(r,s,m)} = \int_{0}^{1} \gamma_{r,s,m}\bigl(\langle x \rangle \bigr)e^{-2\pi i n x}\,dx = \int_{0}^{1} \gamma_{r,s,m}(x)e^{-2\pi i n x} \,dx. $$

To proceed, we need to observe the following. We have

4.4 $$\begin{aligned} &\gamma_{r,s,m}'(x) \\ &\quad =\sum_{c_{1} + \cdots+ c_{r} + j_{1} + \cdots+ j_{s} = m} \frac{1}{c_{2} \cdots c_{r} j_{1} \cdots j_{s}} B_{c_{1}-1}(x)B_{c_{2}}(x) \cdots B_{c_{r}}(x)G_{j_{1}}(x)\cdots G_{j_{s}}(x) \\ &\qquad {} + \cdots \\ &\qquad {}+ \sum_{c_{1} + \cdots+ c_{r} + j_{1} + \cdots+ j_{s} = m} \frac{1}{c_{1} \cdots c_{r-1} j_{1} \cdots j_{s}} B_{c_{1}}(x) \cdots B_{c_{r-1}}(x) B_{c_{r}-1}(x)G_{j_{1}}(x)\cdots G_{j_{s}}(x) \\ &\qquad {} + \sum_{c_{1} + \cdots+ c_{r} + j_{1} + \cdots+ j_{s} = m} \frac{1}{c_{1} \cdots c_{r} j_{2} \cdots j_{s}} B_{c_{1}}(x) \cdots B_{c_{r}}(x)G_{j_{1}-1}(x)G_{j_{2}}(x) \cdots G_{j_{s}}(x) \\ &\qquad {} + \cdots \\ &\qquad {}+ \sum_{c_{1} + \cdots+ c_{r} + j_{1} + \cdots+ j_{s} = m} \frac{1}{c_{1} \cdots c_{r} j_{1} \cdots j_{s-1}} B_{c_{1}}(x) \cdots B_{c_{r}}(x) G_{j_{1}}(x)\cdots G_{j_{s-1}}(x) G_{j_{s}-1}(x) \\ &\quad =\sum_{c_{2} + \cdots+ c_{r} + j_{1} + \cdots+ j_{s} = m-1} \frac{1}{c_{2} \cdots c_{r} j_{1} \cdots j_{s}} B_{c_{2}}(x) \cdots B_{c_{r}}(x)G_{j_{1}}(x)\cdots G_{j_{s}}(x) \\ &\qquad {} + \sum_{c_{1} + \cdots+ c_{r} + j_{1} + \cdots+ j_{s} = m-1} \frac{1}{c_{2} \cdots c_{r} j_{1} \cdots j_{s}} B_{c_{1}}(x) \cdots B_{c_{r}}(x)G_{j_{1}}(x)\cdots G_{j_{s}}(x) \\ &\qquad {} + \cdots \\ &\qquad {}+ \sum_{c_{1} + \cdots+ c_{r-1} + j_{1} + \cdots+ j_{s} = m-1} \frac {1}{c_{1} \cdots c_{r-1}j_{1} \cdots j_{s}} B_{c_{1}}(x) \cdots B_{c_{r-1}}(x)G_{j_{1}}(x)\cdots G_{j_{s}}(x) \\ &\qquad {} + \sum_{c_{1} + \cdots+ c_{r} + j_{1} + \cdots+ j_{s} = m-1} \frac{1}{c_{1} \cdots c_{r-1} j_{1} \cdots j_{s}} B_{c_{1}}(x) \cdots B_{c_{r}}(x)G_{j_{1}}(x)\cdots G_{j_{s}}(x) \\ &\qquad {} + \sum_{c_{1} + \cdots+ c_{r} + j_{2} + \cdots+ j_{s} = m-1} \frac{1}{c_{1} \cdots c_{r} j_{2} \cdots j_{s}} B_{c_{1}}(x) \cdots B_{c_{r}}(x)G_{j_{2}}(x)\cdots G_{j_{s}}(x) \\ &\qquad {} + \sum_{c_{1} + \cdots+ c_{r} + j_{1} + \cdots+ j_{s} = m-1} \frac{1}{c_{1} \cdots c_{r} j_{2} \cdots j_{s}} B_{c_{1}}(x) \cdots B_{c_{r}}(x)G_{j_{1}}(x)\cdots G_{j_{s}}(x) \\ & \qquad {}+ \cdots \\ &\qquad {}+ \sum_{c_{1} + \cdots+ c_{r} + j_{1} + \cdots+ j_{s-1} = m-1} \frac {1}{c_{1} \cdots c_{r}j_{1} \cdots j_{s-1}} B_{c_{1}}(x) \cdots B_{c_{r}}(x)G_{j_{1}}(x)\cdots G_{j_{s-1}}(x) \\ &\qquad {}+ \sum_{c_{1} + \cdots+ c_{r} + j_{1} + \cdots+ j_{s} = m-1} \frac {1}{c_{1} \cdots c_{r}j_{1} \cdots j_{s-1}} B_{c_{1}}(x) \cdots B_{c_{r}}(x)G_{j_{1}}(x)\cdots G_{j_{s}}(x) \\ &\quad = r \gamma_{r-1,s,m-1}(x)+ s \gamma_{r,s-1,m-1}(x) \\ &\qquad {}+ \sum_{c_{1} + \cdots+ c_{r} + j_{1} + \cdots+ j_{s} = m-1} \biggl\{ \frac {1}{c_{2} c_{3} \cdots c_{r} j_{1} \cdots j_{s}} + \cdots+ \frac{1}{c_{1} \cdots c_{r-1}j_{1} \cdots j_{s}} \\ &\qquad {} + \frac{1}{c_{1} \cdots c_{r} j_{2} \cdots j_{s}} + \cdots+ \frac{1}{c_{1} \cdots c_{r}j_{1} \cdots j_{s-1}} \biggr\} B_{c_{1}}(x) \cdots B_{c_{r}}(x)G_{j_{1}}(x)\cdots G_{j_{s}}(x) \\ &\quad = r \gamma_{r-1,s,m-1}(x)+ s \gamma_{r,s-1,m-1}(x) + (m-1)\gamma _{r,s,m-1}(x). \end{aligned}$$

Thus, we have shown that

4.5 $$ \gamma_{r,s,m}'(x) = r \gamma_{r-1,s,m-1}(x)+ s \gamma _{r,s-1,m-1}(x) + (m-1)\gamma_{r,s,m-1}(x). $$

Let $m \geq r+s$, for $r > 0$, and let $m > s$, for $r = 0$. Then we put

4.6 $$ \begin{aligned}[b] \Lambda_{r,s,m} & = \gamma_{r,s,m}(1) - \gamma_{r,s,m}(0) \\ & = \sum_{c_{1} + \cdots+ c_{r} + j_{1} + \cdots+ j_{s} = m} \frac{1}{c_{1} \cdots c_{r} j_{1} \cdots j_{s}} \bigl(B_{c_{1}}(1)\cdots B_{c_{r}}(1)G_{j_{1}}(1)\cdots G_{j_{s}}(1) \\ &\quad {} - B_{c_{1}}\cdots B_{c_{r}}G_{j_{1}}\cdots G_{j_{s}} \bigr) \\ & = \sum_{c_{1} + \cdots+ c_{r} + j_{1} + \cdots+ j_{s} = m} \frac{1}{c_{1} \cdots c_{r} j_{1} \cdots j_{s}} \bigl((B_{c_{1}} + \delta_{1,c_{1}}) \cdots (B_{c_{r}} + \delta_{1,c_{r}}) \\ &\quad {}\times (- G_{j_{1}} + 2\delta_{1,j_{1}})\cdots(- G_{j_{s}} + 2 \delta _{1,j_{s}}) - B_{c_{1}}\cdots B_{c_{r}}G_{j_{1}} \cdots G_{j_{s}} \bigr) \\ & = \sum_{ 0 \leq a \leq r, 0 \leq c \leq s} \binom{r}{a} \binom {s}{c}(-1)^{c} 2^{s-c} \\ &\quad {}\times\sum_{c_{1} + \cdots+ c_{a} + j_{1} + \cdots+ j_{c} = m+a+c-r-s} \frac{1}{c_{1} \cdots c_{a} j_{1} \cdots j_{c}}B_{c_{1}} \cdots B_{c_{a}}G_{j_{1}}\cdots G_{j_{c}} \\ & \quad {}- \sum_{c_{1} + \cdots+ c_{r} + j_{1} + \cdots+ j_{s} = m} \frac{1}{c_{1} \cdots c_{r} j_{1} \cdots j_{s}} B_{c_{1}} \cdots B_{c_{r}}G_{j_{1}}\cdots G_{j_{s}}. \end{aligned} $$

From (4.5), we obtain

4.7 $$ \gamma_{r,s,m}(x) = - \frac{r}{m} \gamma_{r-1,s,m}(x) - \frac{s}{m} \gamma_{r,s-1,m}(x) + \frac{1}{m} \gamma_{r,s,m+1}'(x). $$

Denoting $\int_{0}^{1}\gamma_{r,s,m}(x)\,dx $ by $a_{r,s,m}$, from (4.7) we have

4.8 $$\begin{aligned}& a_{r,s,m} = - \frac{r}{m} a_{r-1,s,m} - \frac{s}{m} a_{r,s-1,m} + \frac{1}{m} \Lambda_{r,s,m+1}, \end{aligned}$$

4.9 $$\begin{aligned}& a_{r,0,m} = \int_{0}^{1}\gamma_{r,0,m}(x)\,dx = \sum _{j=1}^{r} \frac {(-1)^{j-1}(r)_{j-1}}{m^{j}} \Lambda_{r-j+1,0,m+1} \quad( r \geq1), \end{aligned}$$

4.10 $$\begin{aligned}& a_{0,s,m} = \int_{0}^{1}\gamma_{0,s,m}(x)\,dx = \frac{1}{m} \Lambda _{0,s,m+1} \quad( s \geq1). \end{aligned}$$

Clearly, (4.8) together with (4.9) and (4.10) determines $a_{r,s,m}=\int_{0}^{1}\gamma_{r,s,m}(x)\,dx $ recursively for all *r*, *s*, *m* satisfying $m \geq r+s$ if $r > 0$ or $m > s$ if $r = 0$.

Also, we note that

4.11 $$ \gamma_{r,s,m}(0) = \gamma_{r,s,m}(1) \quad \Leftrightarrow\quad \Lambda _{r,s,m} =0. $$

We now would like to determine the Fourier coefficients $C_{n}^{(r,s,m)}$.

*Case 1*: $n\neq0$. We have

4.12 $$ \begin{aligned}[b] C_{n}^{(r,s,m)}&= \int_{0}^{1}\gamma_{r,s,m}(x)e^{-2\pi i n x} \,dx \\ &=-\frac{1}{2\pi i n} \bigl[\gamma_{r,s,m}(x)e^{-2\pi i n x} \bigr]_{0}^{1} +\frac{1}{2\pi i n} \int_{0}^{1}\gamma'_{r,s,m}(x)e^{-2\pi i n x} \, dx \\ &=-\frac{1}{2\pi i n} \bigl(\gamma_{r,s,m}(1)- \gamma_{r,s,m}(0) \bigr) +\frac{1}{2\pi i n} \int_{0}^{1} \bigl\{ r \gamma_{r-1,s,m-1}(x)+ s \gamma_{r,s-1,m-1}(x) \\ &\quad {} + (m-1)\gamma_{r,s,m-1}(x) \bigr\} e^{-2\pi i n x}\,dx \\ &= - \frac{1}{2 \pi i n} \Lambda_{r,s,m} + \frac{1}{2\pi i n} \bigl(rC_{n}^{(r-1,s,m-1)} + sC_{n}^{(r,s-1,m-1)} + (m-1)C_{n}^{(r,s,m-1)} \bigr) \\ &= \frac{m-1}{2\pi i n}C_{n}^{(r,s,m-1)} + \frac{r}{2\pi i n}C_{n}^{(r-1,s,m-1)} + \frac{s}{2\pi i n}C_{n}^{(r,s-1,m-1)} - \frac {1}{2 \pi i n} \Lambda_{r,s,m} \\ & =\frac{m-1}{2\pi i n} \biggl( \frac{m-2}{2\pi i n} C_{n}^{(r,s,m-2)}+ \frac{r}{2\pi i n}C_{n}^{(r-1,s,m-2)} \\ &\quad {} + \frac {s}{2\pi i n}C_{n}^{(r,s-1,m-2)}- \frac{1}{2 \pi i n} \Lambda_{r,s,m-1} \biggr) \\ &\quad {}+ \frac{r}{2\pi i n}C_{n}^{(r-1,s,m-1)}+ \frac{s}{2\pi i n}C_{n}^{(r,s-1,m-1)} - \frac{1}{2 \pi i n} \Lambda_{r,s,m} \\ &=\frac{(m-1)(m-2)}{(2\pi i n)^{2}}C_{n}^{(r,s,m-2)} + \frac {r(m-1)}{(2\pi i n)^{2}} C_{n}^{(r-1,s,m-2)} \\ &\quad {}+ \frac{r}{2\pi i n}C_{n}^{(r-1,s,m-1)}+ \frac{s(m-1)}{(2\pi i n)^{2}} C_{n}^{(r,s-1,m-2)} + \frac{s}{2\pi i n}C_{n}^{(r,s-1,m-1)}\\ &\quad {} - \frac{m-1}{(2 \pi i n)^{2}} \Lambda_{r,s,m-1} - \frac{1}{2 \pi i n} \Lambda_{r,s,m}\hspace{-10pt} \\ & = \cdots \\ & = \frac{(m-1)_{m-(r+s)}}{(2\pi i n)^{m-(r+s)}} C_{n}^{(r,s,r+s)} + \sum _{j=1}^{m-(r+s)} \frac{r(m-1)_{j-1}}{(2\pi i n)^{j}} C_{n}^{(r-1,s,m-j)} \\ &\quad {} + \sum_{j=1}^{m-(r+s)} \frac{s(m-1)_{j-1}}{(2\pi i n)^{j}} C_{n}^{(r,s-1,m-j)} - \sum_{j=1}^{m-(r+s)} \frac{(m-1)_{j-1}}{(2\pi i n)^{j}} \Lambda _{r,s,m-j+1} \\ & = \sum_{j=1}^{m-(r+s)+1} \frac{r(m-1)_{j-1}}{(2\pi i n)^{j}} C_{n}^{(r-1,s,m-j)} + \sum_{j=1}^{m-(r+s)} \frac{s(m-1)_{j-1}}{(2\pi i n)^{j}} C_{n}^{(r,s-1,m-j)} \\ &\quad {} - \sum_{j=1}^{m-(r+s)+1} \frac{(m-1)_{j-1}}{(2\pi i n)^{j}} \Lambda _{r,s,m-j+1}. \end{aligned} $$

Note that

4.13 $$ \begin{aligned} & \frac{(m-1)_{m-(r+s)}}{(2\pi i n)^{m-(r+s)}} C_{n}^{(r,s,r+s)}\\ &\quad = \frac{(m-1)_{m-(r+s)}}{(2\pi i n)^{m-(r+s)}} \int_{0}^{1}\biggl(x -\frac {1}{2} \biggr)^{r} \,dx \\ &\quad = \frac{(m-1)_{m-(r+s)}}{(2\pi i n)^{m-(r+s)}} \biggl\{ -\frac {1}{2\pi i n} \biggl( \biggl( \frac{1}{2}\biggr)^{r} - \biggl(-\frac{1}{2} \biggr)^{r} \biggr)\\ &\qquad {} + \frac{r}{2\pi i n} \int_{0}^{1} \biggl(x -\frac{1}{2} \biggr)^{r-1} e^{-2\pi i n x}\,dx \biggr\} , \\ & \frac{r(m-1)_{m-(r+s)}}{(2\pi i n)^{m-(r+s)+1}} C_{n}^{(r-1,s,r+s-1)} = \frac{r(m-1)_{m-(r+s)}}{(2\pi i n)^{m-(r+s)+1}} \int_{0}^{1} \biggl(x -\frac{1}{2} \biggr)^{r-1} e^{-2\pi i n x}\,dx, \\ & - \frac{(m-1)_{m-(r+s)}}{(2\pi i n)^{m-(r+s)+1}} \Lambda_{r,s,r+s} = - \frac{(m-1)_{m-(r+s)}}{(2\pi i n)^{m-(r+s)+1}} \biggl( \biggl(\frac {1}{2} \biggr)^{r} - \biggl(-\frac{1}{2}\biggr)^{r} \biggr). \end{aligned} $$

Thus we have shown that

4.14 $$\begin{aligned}& \begin{aligned}[b] C_{n}^{(r,s,m)} & = \sum_{j=1}^{m-(r+s)+1} \frac{r(m-1)_{j-1}}{(2\pi i n)^{j}} C_{n}^{(r-1,s,m-j)} + \sum_{j=1}^{m-(r+s)} \frac{s(m-1)_{j-1}}{(2\pi i n)^{j}} C_{n}^{(r,s-1,m-j)} \\ &\quad {} - \sum_{j=1}^{m-(r+s)+1} \frac{(m-1)_{j-1}}{(2\pi i n)^{j}} \Lambda _{r,s,m-j+1}, \end{aligned} \end{aligned}$$

4.15 $$\begin{aligned}& C_{n}^{(r,0,m)} = \sum _{j=1}^{m-r+1} \frac{r(m-1)_{j-1}}{(2\pi i n)^{j}} C_{n}^{(r-1,0,m-j)} - \sum_{j=1}^{m-r+1} \frac{(m-1)_{j-1}}{(2\pi i n)^{j}} \Lambda _{r,0,m-j+1} \quad(r \geq2), \end{aligned}$$

4.16 $$\begin{aligned}& C_{n}^{(1,0,m)} = - \frac{(m-1)!}{(2\pi i n)^{m}}, \end{aligned}$$

4.17 $$\begin{aligned}& C_{n}^{(0,s,m)} = - \frac{1}{m} \sum _{j=1}^{m-s} \frac {(m)_{j}}{(2\pi i n)^{j}} \Lambda_{0,s,m-j+1} \quad(s \geq1). \end{aligned}$$

We now see that $C_{n}^{(r,s,m)}$ ($n \neq0$) can be completely determined by (4.14)-(4.17), for all *r*, *s*, *m* satisfying $m \geq r+s$ if $r > 0$ or $m > s$ if $r = 0$.

*Case 2*: $n = 0 $. We have

4.18 $$ C_{0}^{(r,s,m)}= \int_{0}^{1} \gamma_{r,s,m}(x) \,dx, $$

which can be determined from (4.8)-(4.10), for all *r*, *s*, *m* satisfying $m \geq r+s$ if $r > 0$ or $m > s$ if $r = 0$.

$\gamma_{r,s,m}(\langle x\rangle)$ is piecewise $C^{\infty}$. Moreover, $\gamma_{r,s,m}(\langle x\rangle)$ is continuous for those integers *r*, *s*, *m* with $\Lambda_{r,s,m} = 0$ and discontinuous with jump discontinuities at integers for those integers *r*, *s*, *m* with $\Lambda_{r,s,m} \neq0$.

Assume first that $\Lambda_{r,s,m} = 0 $, for some integers *r*, *s*, *m* satisfying $m \geq r+s$ if $r > 0$ or $m > s$ if $r = 0$. Then $\gamma _{r,s,m}(0)=\gamma_{r,s,m}(1)$. Thus $\gamma_{r,s,m}(\langle x\rangle )$ is piecewise $C^{\infty}$ and continuous. Hence the Fourier series of $\gamma_{r,s,m}(\langle x\rangle)$ converges uniformly to $\gamma _{r,s,m}(\langle x\rangle)$, and

$$\gamma_{r,s,m}\bigl(\langle x\rangle\bigr) = C_{0}^{(r,s,m)} + \sum_{n=-\infty ,n\neq0}^{\infty}C_{n}^{(r,s,m)}e^{2\pi i n x}, $$

where $C_{0}^{(r,s,m)}$ are determined by (4.8)-(4.10) and $C_{n}^{(r,s,m)}$ ($n \neq0$) by (4.14)-(4.17).

We are now going to state our first result.

### Theorem 4.1

*For all integers* *r*, *s*, *l* *satisfying* $l \geq r+s$, *for* $r > 0$ *or* $l > s$, *for* $r = 0$, *we let*

$$ \begin{aligned}[b] \Lambda_{r,s,l} & = \sum _{ 0 \leq a \leq r, 0 \leq c \leq s} \binom {r}{a} \binom{s}{c}(-1)^{c} 2^{s-c} \\ &\quad {} \times\sum_{c_{1} + \cdots+ c_{a} + j_{1} + \cdots+ j_{c} = l+a+c-r-s} \frac{1}{c_{1} \cdots c_{a} j_{1} \cdots j_{c}}B_{c_{1}} \cdots B_{c_{a}}G_{j_{1}}\cdots G_{j_{c}} \\ &\quad {} - \sum_{c_{1} + \cdots+ c_{r} + j_{1} + \cdots+ j_{s} = l} \frac{1}{c_{1} \cdots c_{r} j_{1} \cdots j_{s}} B_{c_{1}} \cdots B_{c_{r}}G_{j_{1}}\cdots G_{j_{s}}. \end{aligned} $$

*Assume that* $\Lambda_{r,s,m} = 0 $, *for some integers* *r*, *s*, *m* *satisfying* $m \geq r+s$ *if* $r > 0$ *or* $m > s$ *if* $r = 0$. *Then we have the following*:

$$ \begin{aligned}[b] &\sum_{c_{1} + \cdots+ c_{r} + j_{1} + \cdots+ j_{s} = m} \frac{1}{c_{1} \cdots c_{r} j_{1} \cdots j_{s}} B_{c_{1}}\bigl(\langle x\rangle\bigr)\cdots B_{c_{r}}\bigl(\langle x\rangle\bigr) \\ &\quad {}\times G_{j_{1}}\bigl(\langle x\rangle\bigr)\cdots G_{j_{s}} \bigl(\langle x\rangle\bigr) \end{aligned} $$

*has the Fourier series expansion*

$$ \begin{aligned}[b] & \sum_{c_{1} + \cdots+ c_{r} + j_{1} + \cdots+ j_{s} = m} \frac{1}{c_{1} \cdots c_{r} j_{1} \cdots j_{s}} B_{c_{1}}\bigl(\langle x\rangle\bigr)\cdots B_{c_{r}}\bigl(\langle x\rangle\bigr) \\ &\qquad {}\times G_{j_{1}}\bigl(\langle x\rangle\bigr)\cdots G_{j_{s}} \bigl(\langle x\rangle\bigr) \\ &\quad = C_{0}^{(r,s,m)} + \sum_{n=-\infty,n\neq0}^{\infty }C_{n}^{(r,s,m)}e^{2\pi i n x}, \end{aligned} $$

*where* $C_{n}^{(r,s,m)}$ ($n \neq0$) *are determined by* (4.14)-(4.17) *and* $C_{0}^{(r,s,m)}$ *by* (4.8)-(4.10). *Here the convergence is uniform*.

Next, assume that $\Lambda_{r,s,m} \neq0 $, for some integers satisfying $m \geq r+s$ if $r > 0$ or $m > s$ if $r = 0$. Then $\gamma _{r,s,m}(0) \neq\gamma_{r,s,m}(1)$. Here $\gamma_{r,s,m}(\langle x\rangle)$ is piecewise $C^{\infty}$ and discontinuous with jump discontinuities at integers. Then the Fourier series of $\gamma_{r,s,m}(\langle x\rangle)$ converges pointwise to $\gamma_{r,s,m}(\langle x\rangle )$, for $x \notin\mathbb{Z}$, and it converges to

4.19 $$ \frac{1}{2}\bigl(\gamma_{r,s,m}(0)+ \gamma_{r,s,m}(1)\bigr)=\gamma_{r,s,m}(0) + \frac{1}{2} \Lambda_{r,s,m}, $$

for $x\in\mathbb{Z}$.

Now, we are going to state our second result.

### Theorem 4.2

*For all integers* *r*, *s*, *l* *satisfying* $l \geq r+s$, *for* $r > 0$ *or* $l > s$, *for* $r = 0$, *we let*

$$ \begin{aligned}[b] \Lambda_{r,s,l} & = \sum _{ 0 \leq a \leq r, 0 \leq c \leq s} \binom {r}{a} \binom{s}{c}(-1)^{c} 2^{s-c} \\ &\quad \times{} \sum_{c_{1} + \cdots+ c_{a} + j_{1} + \cdots+ j_{c} = l+a+c-r-s} \frac {1}{c_{1} \cdots c_{a} j_{1} \cdots j_{c}}B_{c_{1}} \cdots B_{c_{a}}G_{j_{1}}\cdots G_{j_{c}} \\ &\quad {} - \sum_{c_{1} + \cdots+ c_{r} + j_{1} + \cdots+ j_{s} = l} \frac{1}{c_{1} \cdots c_{r} j_{1} \cdots j_{s}} B_{c_{1}} \cdots B_{c_{r}}G_{j_{1}}\cdots G_{j_{s}}. \end{aligned} $$

*Assume that* $\Lambda_{r,s,m} \neq0 $, *for some integers* *r*, *s*, *m* *satisfying* $m \geq r+s$ *if* $r > 0$ *or* $m > s$ *if* $r = 0$. *Then we have the following*:

$$ \begin{aligned}[b] &C_{0}^{(r,s,m)} + \sum _{n=-\infty,n\neq0}^{\infty }C_{n}^{(r,s,m)}e^{2\pi i n x} \\ &\quad = \textstyle\begin{cases} \sum_{c_{1} + \cdots+ c_{r} + j_{1} + \cdots+ j_{s} = m} \frac{1}{c_{1} \cdots c_{r} j_{1} \cdots j_{s}} B_{c_{1}}(\langle x\rangle)\cdots B_{c_{r}}(\langle x\rangle)\\ \quad {}\times G_{j_{1}}(\langle x\rangle)\cdots G_{j_{s}}(\langle x\rangle),& \textit{for } x \notin\mathbb{Z},\\ \sum_{c_{1} + \cdots+ c_{r} + j_{1} + \cdots+ j_{s} = m} \frac{1}{c_{1} \cdots c_{r} j_{1} \cdots j_{s}} B_{c_{1}}\cdots B_{c_{r}}G_{j_{1}}\cdots G_{j_{s}} + \frac{1}{2} \Lambda_{r,s,m},& \textit{for } x \in\mathbb{Z}, \end{cases}\displaystyle \end{aligned} $$

*where* $C_{n}^{(r,s,m)}$ ($n \neq0$) *are determined by* (4.14)-(4.17) *and* $C_{0}^{(r,s,m)}$ *by* (4.8)-(4.10).

## Results and discussion

In this paper, we study three types of functions which are given by products of Bernoulli and Genocchi functions and we give some new identities arising from Fourier series expansions associated with Bernoulli and Genocchi functions. In addition, we will express each of them in terms of Bernoulli functions. The Fourier series expansion of the Bernoulli and Genocchi functions are useful in computing the special values of the zeta and multiple zeta function. It is expected that the Fourier series of the Bernoulli and Genocchi functions will find some applications in connection with a certain zeta function and the higher-order Bernoulli numbers.

## Conclusion

In this paper, we considered the Fourier series expansion of the Bernoulli and Genocchi functions which are obtained by extending by periodicity of period the Bernoulli and Genocchi polynomials on $[0, 1)$. The Fourier series are explicitly determined.
